# Genetic variations in MAGE-A11 predict the risk and survival of renal cell cancer

**DOI:** 10.7150/jca.32675

**Published:** 2019-08-27

**Authors:** Shifeng Su, Qi Gu, Aiming Xu, Sipeng Shen, Shouyong Liu, Chao Zhang, Chengkui Miao, Chao Qin, Bianjiang Liu, Zengjun Wang

**Affiliations:** 1 Department of Urology, The First Affiliated Hospital of Nanjing Medical University, Nanjing 210029, China; 2 Department of Biostatistics, School of Public Health, Nanjing Medical University, Nanjing, 211166, Jiangsu, China.; 3 Department of Environmental Health, Harvard T.H. Chan School of Public Health, Harvard University, Boston, MA, USA.

**Keywords:** renal carcinoma, MAGE-A11, single-nucleotide polymorphism, survival

## Abstract

Melanoma antigen-A11 (*MAGE-A11*) is a low-abundance, primate-specific steroid receptor coregulator in normal tissues of the human reproductive tract, which plays an important role in tumorigenesis. Single-nucleotide polymorphisms (SNPs) have been shown to contribute to cancer risk and prognosis. However, the role of SNPs of *MAGE-A11* in renal cell carcinoma (RCC) has not been established. Two intronic SNPs (rs6641352 and rs6540341) of *MAGE-A11* have been screened to assess their associations with RCC risk and prognosis in a case control study. We found that rs6641352 was associated with RCC susceptibility in the dominant model (TC/CC vs. TT, adjusted odds ratio = 1.315, 95% confidence interval [CI] = 1.089-1.588) and with survival of RCC in the recessive model (CC vs. TT/TC, adjusted hazard ratio = 3.526, 95% CI = 1.072-11.595). For the SNP rs6540341, individuals with the T allele could have a critically increased risk of RCC (adjusted odds ratio = 1.301, 95% CI = 1.081-1.564, *P* = 0.005 in the dominant model). However, there was no significant association between rs6540341 and RCC survival. Hence, rs6641352 in *MAGE-A11* may contribute to the genetic susceptibility and prognosis for RCC and act as a biomarker for RCC occurrence and prognosis.

## Introduction

Renal cell carcinoma is the most common lethal tumor of all the urological neoplasms 1. There is a total of 209,000 new cases and 102,000 deaths per year worldwide, with the male-to-female ratio being 1.5:1.0, with peaks at age 60-70 years 2. The specific causes of RCC are still unknown, but epidemiological studies have reported that many factors, like smoking tobacco, hypertension, kidney diseases, diabetes, obesity, and genetics, could increase the risk of RCC 3-5.

The single-nucleotide polymorphism (SNP) is the simplest form of DNA variation among individuals. Some SNPs in coding regions change the amino acid sequence of a protein, and others in the coding region do not affect the protein sequence. SNPs outside the coding region may also affect transcription factor binding, gene splicing, or mRNA degradation 6. Many studies have reported that SNPs can act as genetic markers to identify the complete set of genes that are involved in the development of renal cancer. For example, two genetic susceptibility loci, rs4415084 and rs10941679, at chromosome 5p12 are associated with breast cancer risk 7, while two SNPs significantly associated with miRNA expression levels, rs8176318 (BRCA1) and rs8905 (PRKAR1A), are associated with colon cancer risk 8.

Melanoma antigen-A11 (*MAGE-A11*) belongs to the *MAGE-A* subfamily of cancer-germline antigens at the Xq28 locus of the human X chromosome 9. *MAGE-A11*, which specifically binds to the human androgen receptor (AR) N-terminal FXXLF motif and functions as an AR coregulator that increases transcriptional activity of AR, competing with the AR N/C interaction and exposing the activation function 2 site in the ligand binding domain 10. *MAGE-A11* has a low expression level in normal human testis, ovary, and placenta, while it is upregulated during prostate cancer progression due to hypomethylation of the *MAGE-A11* promoter and increasing cyclic AMP levels, associated with increased AR transcriptional activity 11, 12. Our previous study also found that *MAGE-A11* and AR cooperated in the upregulation of FSTL1 to promote growth and progression of castration-resistant/recurrent prostate cancer 13. However, to the best of our knowledge, the role of *MAGE-A11* in RCC has not been reported.

In this study, we selected two common SNPs of the *MAGE-A11* gene (i.e., S1.rs6641352 T>C and S2.rs6540341 C>T) to evaluate their associations with the risk and survival of RCC by a two-stage case control study and a cohort study. Further analyses were conducted to determine the effects of SNPs on RCC and survival.

## Materials and methods

### Selection and characteristics of patients

The project was approved by the Institutional Review Board of the Nanjing Medical University. Each participant involved in this study signed a written informed consent prior to inclusion in the study. A total of 1027 cases and 1094 controls were collected from May 2004 to March 2014 at the First Affiliated Hospital of Nanjing Medical University. The inclusion criteria have been described previously 14, 15: (1) The cases were newly diagnosed with incident RCC. (2) The cases had been histopathologically confirmed. (3) The cases did not have a prior history of other malignancies. (4) The cases have not been treated with chemotherapy or radiotherapy. (5) The cases have complete treatment and follow-up information. The controls were recruited from subjects without any individual history of cancer who were seeking health care in the outpatient departments at the hospital and were frequency matched to the cases for sex and age (±5 years). In this cohort, 17 patients and 140 controls were excluded due to low DNA concentrations or because of incomplete data. Each patient's RCC classification and stage were determined according to the TNM staging system by the American Joint Committee on Cancer (AJCC). The validation cohort was made up sampling randomly 500 cases and 470 controls from subjects conforming the inclusion criteria the by IBM SPSS 24.0. All enrolled patients were frequency-matched for age (±5 years) and sex. For the survival analysis, 355 patients were followed up prospectively for overall survival information every 6 months from the histological confirmation until death or the last follow-up. Of them, 47 patients were excluded due to low DNA concentrations or a lack of complete follow-up information.

### SNP selection and genotyping

We identified potentially functional polymorphisms according to the following criteria: (1) located in the 5′ flanking region, the 5′ untranslated region (UTR), the 3′-UTR, or the coding region causing an amino acid change; (2) minor allele frequency (MAF) > 0.05 in the CHB and JPT population from the 1000 Genomes Project; (3) *r^2^* > 0.8 based on the pairwise linkage disequilibrium using Haploview version 4.2. Two polymorphisms in *MAGE-A11* (rs6641352 and rs6540341) were selected for further analysis and processing. Genotyping was performed using the TaqMan SNP genotyping method, as previously described 14.

### Statistical analysis

Differences in the distribution of selected demographic variables between RCC cases and cancer-free controls were evaluated using the Student *t* test for continuous variables and Pearson's χ^2^ test or Fisher's exact test for categorical variables. The Hardy-Weinberg equilibrium for all SNP allele frequencies among controls was tested using a goodness-of-fit χ^2^ test. The associations between SNPs and RCC susceptibility were estimated by computing odds ratios and 95% confidence intervals (CIs) from unconditional logistic regression analyses. Bonferroni correction was applied for multiple comparison. Four genetic models (additive, dominant, recessive, and codominant) were used to assess the effects of SNPs. The heterogeneity between subgroups was estimated with the χ^2^ based on the Q-test. The survival time curves were estimated using the Kaplan-Meier method, and comparisons were made by the log-rank test. Survival time was calculated from the date of RCC diagnosis to the date of death or the last follow-up. Cox proportional hazard models were used to calculate hazard ratios and 95% CIs for predicting factors of RCC survival. A *P*-value <0.05 is considered statistically significant. All statistical analyses were performed by IBM SPSS 24.0. The survival plot was performed by GraphPad Prism 7.

## Results

### Characteristics of study population

The demographic characteristics and clinical features of RCC patients and controls in totality and validation set are listed in Table 1 and S1, respectively. No significant differences were found among patients and controls in terms of age, body mass index, gender, smoking status, drinking status, and family history of cancer (all *P* > 0.05). However, more hypertension and diabetes were observed in patients than in controls (both *P* < 0.001), suggesting that hypertension and diabetes may contribute to RCC development.

### Associations between the MAGE-A11 SNPs and RCC risk

The characteristics of the selected SNPs are presented in Table 2. All genotype frequencies of SNPs conformed to the Hardy-Weinberg equilibrium (0.472 and 0.467, respectively). As shown in Table 3, both selected SNPs were significantly associated with RCC risk. For rs6641352 in the gene* MAGE-A11*, individuals with the C allele had a higher risk of tumorigenesis (odds ratio = 1.315, 95% CI = 1.089-1.588, *P* = 0.004 in the dominant model). Significant associations were also observed in the additive model (odds ratio = 1.250, 95% CI = 1.069-1.461, *P* = 0.005), even after the Bonferroni correction (*P* = 0.020). However, the significance of rs6641352 disappeared in the validation set after Bonferroni correction (*P* = 0.068). No obvious significance was found in the recessive model (*P* = 0.220).

Upon our stratified analysis of individual characteristics and clinicopathological features (Tables S2 and S3), we detected a pathogenic effect of the rs6641352 C allele among subjects with lower age and body mass index (BMI), with smoking and hypertension, without drinking, diabetes, and a family history of cancer, and among the male and patients in an early stage (all *P* < 0.02). However, there was no association between the rs6641352 genotype and clinical features (Table S4).

For the SNP rs6540341, a similar effect on kidney tumorigenesis was found. As shown in Table 3, the genotypes CT/TT could increase the risk of RCC occurrence compared with the homozygous CC genotype (odds ratio = 1.301, 95% CI = 1.081-1.564, *P* < 0.001 in the dominant model), which was confirmed by the validation set(odds ratio = 1.317, 95% CI = 1.014-1.711, *P* = 0.039). We further conducted the stratified analysis and found a negative effect of the rs6540341 T allele among those with lower age and higher body mass index, without drinking, hypertension, and family history of cancer and among the females. The same consequence was observed among patients at an early stage and grade and pathologically diagnosed with RCC (all *P* ≤ 0.05; Tables S5 and S6). However, there was no association between the rs6540341 genotype and clinical features (Table S7).

### Effects of two SNPs on RCC survival

**Figure 1:**
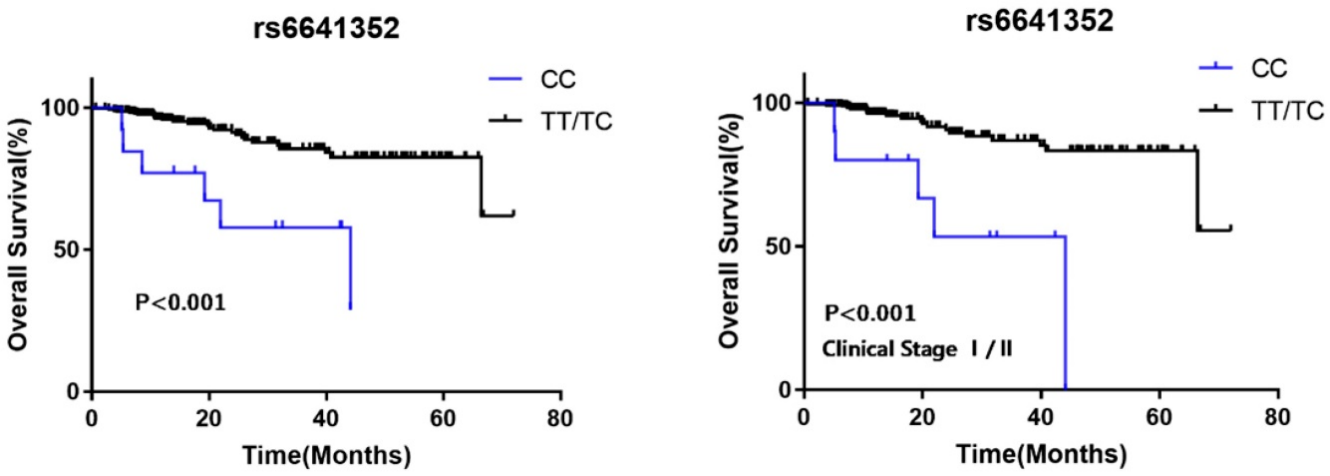
Kaplan-Meier analysis for MAGE-A11 rs6641352 polymorphism genotype patients with RCC. (A)Survival curve for RCC patients by MAGE-A11 rs6641352 genotype in recessive model. (B) Stage-specific Kaplan-Meier survival curve for RCC clinical stage I/II patients with different genotypes.

To explore the effects of the two SNPs on RCC survival, we analyzed clinical follow-up data of 308 RCC patients. The average follow-up time was 14.9 months (ranging from 0.63 to 72 months). For rs6641352, as shown in Table 3 and Figure 1, no patients with the rare homozygous CC genotype lived to 5 years, suggesting a poorer prognosis compared with those with the T allele (hazard ratio = 3.526, 95% CI = 1.072-11.595; log-rank *P* < 0.001), especially in stage I/II (log-rank *P* < 0.001). The results of the advanced stage are questionable due to the small sample size.

The characteristics and clinical features of RCC patients are listed in Table S8. Due to the fact that the small number of individuals with rs6641352CC is further reduced in the stratified analysis, which may cause unstable associations, we will not discuss the stratified analysis of *MAGE-A11* rs6641352, though the results are presented in Table S9. Stepwise Cox proportional hazard analysis was carried out for further analysis (Table S10); seven variables, including rs6641352 in the recessive model, were retained in the regression model, indicating that rs6641352 may be an independent prognosis factor. For rs6540341, there was no association observed in either of the four genetic models (Table S11).

## Discussion

In a previous study, we found that in prostate cancer, *MAGE-A11* is a proto-oncogene, the increased expression of which reverses retinoblastoma-related protein p107 from a transcriptional repressor to a transcriptional activator of the AR and E2F1 16. *MAGE-A11* is a cancer-testis antigen of the* MAGE-A* gene family, notable for its increased expression in cancer 2, 9, 17. Many carcinomas, like breast cancer 18, 19, head and neck carcinomas 20, and laryngeal squamous cell carcinoma 21, have been associated with *MAGE-A11*. However, the role of *MAGE-A11* in RCC has not been reported.

In this study, we evaluated the associations between two SNPs in *MAGE-A11* and RCC susceptibility and prognosis. We found that* MAGE-A11* rs6641352 and rs6540341 are associated with an increased risk of RCC. We also observed a negative impact of rs6641352 on RCC survival, while rs6540341 seemed irrelevant to prognosis.

As regards rs6641352, we observed that the TC/CC genotypes significantly increased the risk of RCC, the heterozygous genotype TC more so than the CC genotype. Further stratification analyses suggested that the association between rs6641352 and the increased RCC risk was more prominent in males, smokers, and hyperpietics, which agrees with epidemiology statistics 3-5. Unexpectedly, subjects without drinking history, diabetes, and family history of cancer showed a stronger susceptibility to RCC. In addition, the CC genotype (vs. TC/TT) of rs6641352 showed a 3.526-fold increased hazard ratio for RCC survival, independently predicting an unfavorable postoperative prognosis in RCC, while we did not obtain statistically significant results for the different alleles of rs6540341.

For rs6540341, all four models showed a strongly increased risk of RCC; especially when considering the additive model or the recessive model, we could hypothesize that the C allele of rs6540341 plays a critical role in renal carcinogenesis. Moreover, the stratification analyses showed a higher risk of RCC among the younger, the obese, and the females, as well as those without hypertension, drinking history, or family history of cancer. The stratification analyses on rs6641352 implied that there may be an interaction between the SNPs and the risk of developing RCC.

The roles of intronic SNPs in tumor formation have received more and more attention in recent years. The intronic SNPs are involved in gene regulation via an intronic enhancer, by regulating expression levels, or by other regulatory modifications 22, 23. As rs6641352 and rs6540341 are intronic SNPs based on genome browser data (http://genome.ucsc.edu; data not shown), they may be passengers rather than drivers in the tumorigenesis of RCC. Both of them are not localized in the predicted regulatory regions of *MAGE-A11*.

In conclusion, this is the first study to explore the epidemiological evidence on *MAGE-A11* SNPs and their statistic relationships with RCC and overall survival rate in the Chinese population. We identified two new loci that are associated with RCC occurrence, *MAGE-A11* rs6641352 and rs6540341, while rs6641352 could also predict RCC patients' survival. However, the data for survival analysis are not sufficient, and the study is lacking an independent cohort for validation. The underlying mechanism by which the two *MAGE-A11* SNPs cause RCC morbidity is still unknown. Further *in vitro* and *in vivo* research is required.

## Supplementary Material

Supplementary tables.

## Figures and Tables

**Table 1:** Demographic characteristic and clinical features among RCC case patients and control subjects.

Characteristics	Number of cases (%)	Number of controls (%)	*P*^a^
Overall Age (years) (mean ± SD)	1010 56.72 ± 12.08	954 55.86 ± 11.61	0.109
BMI (kg/m2) (mean ± SD)	24.07 ± 2.91	23.82 ± 3.27	0.074
Gender			0.093
Male	640 (63.4)	639 (67.0)	
Female	370 (36.6)	315 (33.0)	
Smoking status			0.077
Never	638 (63.2)	639 (67.0)	
Ever	372 (36.8)	315 (33.0)	
Drinking status			0.830
Never	741 (73.4)	704 (73.8)	
Ever	269 (26.6)	250 (26.2)	
**Hypertension**			**<0.001**
No	620 (61.4)	886 (92.9)	
Yes	390 (38.6)	68 (7.1)	
**Diabetes**			**<0.001**
No	879 (87.0)	896 (93.9)	
Yes	131 (13.0)	58 (6.1)	
Family history of cancer			0.602
No	944 (93.5)	886 (92.9)	
Yes	66 (6.5)	68 (7.1)	
Clinical stage			
Ⅰ	221 (21.9)		
Ⅱ	514 (50.9)		
Ⅲ	211 (20.9)		
Ⅳ	64 (6.3)		
Tumor grade			
Ⅰ	658 (65.1)		
Ⅱ	198 (19.6)		
Ⅲ	73 (7.2)		
Ⅳ	81 (8.0)		
Histology			
Clear cell	843 (83.5)		
Papillary	36 (3.6)		
Chromophobe	55 (5.4)		
Unclassified	76 (7.5)		

^a^Student's t-test for age and BMI distributions between cases and controls; χ2-test of R-by-C table for other selected variables between cases and controls.

**Table 2:** Demographic characteristic of 2 SNPs in *MAGE-A11* gene.

SNPs	Alleles (major/minor)	Cases^a^ (n=1010)	Contorls^a^ (n=954)		Location	MAF^b^ (Case/Control)	Call rate (%)	HWE^c^
rs6641352	T/C	596/357/57	625/287/42		3'-UTR	0.233/0.194	98.9	0.472
rs6540341	C/T	561/359/90	586/315/53		3'-UTR	0.267/0.221	98.9	0.467

^a^ Major homozygote/heterozygote/minor homozygote between cases and controls. ^b^ MAF (minor allele frequency) between case and control group. ^c^ HWE (Hardy-Weinberg equilibrium) test among controls.

**Table 3:** Association of the 2 SNPs in *MAGE-A11* gene and risk of RCC in totality and validation set.

Stages	SNPs	OR (95% CI)^a^					P^b^	Bonferroni^c^
		Additive model	Dominant model	Recessive model	Codominant model			
					het	hom		
Totality	rs6641352	**1.250 (1.069-1.461)**	**1.315 (1.089-1.588)**	1.299 (0.855-1.975)	**1.299 (1.066-1.583)**	1.423 (0.931-2.174)	**0.005**	**0.020**
	rs6540341	**1.275 (1.102-1.476)**	**1.301 (1.081-1.564)**	**1.614 (1.123-2.320)**	**1.226 (1.009-1.491)**	**1.740 (1.202-2.519)**	**0.001**	**0.004**
Validation	rs6641352	**1.309 (1.049-1.633)**	**1.456 (1.113-1.905)**	1.112 (0.618-2.001)	**1.489 (1.123-1.974)**	1.269 (0.700-2.302)	**0.017**	0.068
	rs6540341	**1.298 (1.056-1.596)**	**1.317 (1.014-1.711)**	**1.717 (1.030-2.862)**	1.228 (0.931-1.619)	**1.852 (1.100-3.120)**	**0.013**	0.052

Values in bold indicate are statistically different. ^a^Logistic regression model with adjustment for age, sex, BMI, smoking status, drinking status, hypertension, diabetes and family history of cancer in additive (rare homozygote versus heterozygote versus major homozygote) models, dominant (heterozygote/rare homozygote versus major homozygote), recessive (rare homozygote versus heterozygote/major homozygote) and codominant (het: heterozygote versus major homozygote; hom: rare homozygote versus major homozygote). BMI, body mass index; OR, odds ratio. ^b^Adjusted for age, sex, BMI, smoking status, drinking status, hypertension, diabetes and family history of cancer in additive model ^c^Bonferroni correction for additive model.

**Table 4:** Associations between the 2 SNPs in *MAGE-A11* gene and RCC patients' survival.

rs6641352 (T/C)	Patients/deaths	5-year survival (%)^a^	Log-rank P	Crude HR (95% CI)	HR (95% CI)^b^	P^b^
Total number of subjects	308/32	80				
Codominant model						
TT	191/17	81		1.000 (reference)	1.000 (reference)	
TC	104/9	86	0.554	0.803 (0.353-1.823)	0.753 (0.291-1.953)	0.560
CC	13/6	——	**0.001**	**4.193 (1.635-10.757)**	3.217 (0.940-11.013)	0.063
P trend			0.079			
Additive model			**0.001**	1.625 (0.940-2.809)	1.415 (0.773-2.592)	0.261
Dominant model						
TT	191/17	81		1.000 (reference)	1.000 (reference)	
TC/CC	117/15	79	0.653	0.636 (0.584-2.410)	1.110 (0.488-2.523)	0.804
Recessive model						
TT/TC	295/26	83		1.000 (reference)	1.000 (reference)	
CC	13/6	——	**<0.001**	**4.569 (1.870-11.161)**	**3.526 (1.072-11.595)**	**0.038**

BMI, body mass index; HR, hazard ratio. Values in bold indicate statistically different. ^a^Proportion of survival derived from Kaplan-Meier analysis. ^b^Adjusted for age, sex, BMI, smoking status, drinking status, hypertension, diabetes and family history of cancer, clinical stage, tumor stage and histology in Cox regression model.

## References

[1] Rini BI, Campbell SC, Escudier B (2009). Renal cell carcinoma. Lancet (London, England).

[2] De Plaen E, Arden K, Traversari C, Gaforio JJ, Szikora JP, De Smet C (1994). Structure, chromosomal localization, and expression of 12 genes of the MAGE family. Immunogenetics.

[3] Chow WH, Dong LM, Devesa SS (2010). Epidemiology and risk factors for kidney cancer. Nat Rev Urol.

[4] Macleod LC, Hotaling JM, Wright JL, Davenport MT, Gore JL, Harper J (2013). Risk factors for renal cell carcinoma in the VITAL study. J Urol.

[5] Ljungberg B, Campbell SC, Choi HY, Jacqmin D, Lee JE, Weikert S (2011). The epidemiology of renal cell carcinoma. Eur Urol.

[6] Shastry BS (2009). SNPs: impact on gene function and phenotype. Methods Mol Biol.

[7] Liu X, Qin Z, Shen H, Xue J, Jiang Y, Hu Z (2012). Genetic variants at 5p12 and risk of breast cancer in Han Chinese. J Hum Genet.

[8] Mullany LE, Wolff RK, Herrick JS, Buas MF, Slattery ML (2015). SNP Regulation of microRNA Expression and Subsequent Colon Cancer Risk. PLoS One.

[9] Rogner UC, Wilke K, Steck E, Korn B, Poustka A (1995). The melanoma antigen gene (MAGE) family is clustered in the chromosomal band Xq28. Genomics.

[10] Bai S, He B, Wilson EM (2005). Melanoma antigen gene protein MAGE-11 regulates androgen receptor function by modulating the interdomain interaction. Mol Cell Biol.

[11] Karpf AR, Bai S, James SR, Mohler JL, Wilson EM (2009). Increased expression of androgen receptor coregulator MAGE-11 in prostate cancer by DNA hypomethylation and cyclic AMP. Mol Cancer Res.

[12] Artamonova II, Gelfand MS (2004). Evolution of the exon-intron structure and alternative splicing of the MAGE-A family of cancer/testis antigens. J Mol Evol.

[13] Huang Q, Sun Y, Ma X, Gao Y, Li X, Niu Y (2017). Androgen receptor increases hematogenous metastasis yet decreases lymphatic metastasis of renal cell carcinoma. Nat Commun.

[14] Cao Q, Liang C, Xue J, Li P, Li J, Wang M (2016). Genetic variation in IGF1 predicts renal cell carcinoma susceptibility and prognosis in Chinese population. Scientific reports.

[15] Cai H, Sun L, Cui L, Cao Q, Qin C, Zhang G (2013). A functional insertion/deletion polymorphism (-94 ins/del ATTG) in the promoter region of the NFKB1 gene is related to the risk of renal cell carcinoma. Urol Int.

[16] Su S, Minges JT, Grossman G, Blackwelder AJ, Mohler JL, Wilson EM (2013). Proto-oncogene activity of melanoma antigen-A11 (MAGE-A11) regulates retinoblastoma-related p107 and E2F1 proteins. J Biol Chem.

[17] Sang M, Lian Y, Zhou X, Shan B (2011). MAGE-A family: attractive targets for cancer immunotherapy. Vaccine.

[18] Xia LP, Xu M, Chen Y, Shao WW (2013). Expression of MAGE-A11 in breast cancer tissues and its effects on the proliferation of breast cancer cells. Mol Med Rep.

[19] Hou SY, Sang MX, Geng CZ, Liu WH, Lu WH, Xu YY (2014). Expressions of MAGE-A9 and MAGE-A11 in breast cancer and their expression mechanism. Arch Med Res.

[20] Hartmann S, Zwick L, Scheurer MJJ, Fuchs AR, Brands RC, Seher A (2018). MAGE-A11 expression contributes to cisplatin resistance in head and neck cancer. Clin Oral Investig.

[21] Liu S, Sang M, Xu Y, Gu L, Liu F, Shan B (2016). Expression of MAGE-A1, -A9, -A11 in laryngeal squamous cell carcinoma and their prognostic significance: a retrospective clinical study. Acta Otolaryngol.

[22] Yablonovitch AL, Fu J, Li K, Mahato S, Kang L, Rashkovetsky E (2017). Regulation of gene expression and RNA editing in Drosophila adapting to divergent microclimates. Nat Commun.

[23] Ji XF, Chi TY, Xu Q, He XL, Zhou XY, Zhang R (2014). Xanthoceraside ameliorates mitochondrial dysfunction contributing to the improvement of learning and memory impairment in mice with intracerebroventricular injection of abeta1-42. Evid Based Complement Alternat Med.

