# Supplementary material for: Genetic variations in MAGE-A11 predict the risk and survival of renal cell cancer
Source: J Cancer. 2019 Aug 27;10(20):4860–5. doi: 10.7150/jca.32675 (PMC6775520; doi:10.7150/jca.32675)
Supplement: Supplementary file 1 — Supplementary tables. [file jcav10p4860s1.pdf]

Table S1. Demographic characteristic and clinical features among RCC case patients and control subjects in validation set.

Characteristics	Number of cases (%)	Number of controls (%)
Overall	500	470
Age (years) (mean ± SD)	56.53±11.94	55.78±11.83
BMI (kg/m2) (mean ± SD)	24.09±2.85	23.87±3.31
Gender		
Male	316 (63.2)	313 (66.6)
Female	184 (36.8)	157 (33.4)
Smoking status		
Never	321 (64.2)	309 (65.7)
Ever	179 (35.8)	161 (34.3)
Drinking status		
Never	369 (73.8)	344 (73.2)
Ever	131 (26.2)	126 (26.8)
Hypertension		
No	302 (60.4)	347 (73.8)
Yes	198 (39.6)	123 (26.2)
Diabetes		
No	438 (87.6)	437 (93.0)
Yes	62 (12.4)	33 (7.0)
Family history of cancer		
No	466 (93.2)	443 (94.3)
Yes	34 (6.8)	27 (5.7)
Clinical stage		
Ⅰ	324 (64.8)	
Ⅱ	100 (20.0)	
Ⅲ	32 (6.4)	
Ⅳ	44 (8.8)	
Tumor grade		
Ⅰ	106 (21.2)	
Ⅱ	259 (51.8)	
Ⅲ	107 (21.4)	
Ⅳ	28 (5.6)	
Histology		
Clear cell	420 (84.0)	
Papillary	22 (4.4)	
Chromophobe	26 (5.2)	
Unclassified	32 (6.4)	

Table S2. Stratification analyses between *MAGE-A11*rs6641352 polymorphism and risk factors in dominant model.

Variables	Cases [a]	Controls[a]	OR (95%CI)[b]	P[b]
Age				
≤57	308/190/30	366/165/29	**1.338(1.038-1.725)**	**0.025**
＞57	288/167/27	269/15422	1.302(0.977-1.735)	0.071
BMI				
≤24	309/166/36	356/133/20	**1.450(1.108-1.899)**	**0.007**
＞24	287/191/21	269/154/22	1.180(0.903-1.542)	0.226
Gender				
Male	359/239/42	413/196/30	**1.408(1.116-1.778)**	**0.004**
Female	237/118/15	212/91/12	1.169(0.846-1.617)	0.344
Smoking status				
Never	395/213/30	424/184/31	1.205(0.954-1.521)	0.117
Former	88/56/10	41/19/3	1.654(0.863-3.170)	0.13
Current	113/88/17	160/84/8	**1.672(1.137-2.457)**	**0.009**
Drinking status				
Never	448/256/37	473/199/32	**1.324(1.062-1.650)**	**0.013**
Ever	148/101/20	152/88/10	1.361(0.934-1.981)	0.108
Hypertension				
No	372/211/37	454/223/31	1.156(0.921-1.451)	0.221
Yes	224/146/20	171/64/11	**1.830(1.286-2.604)**	**0.001**
Diabetes				
No	525/306/48	591/267/38	**1.325(1.087-1.615)**	**0.005**
Yes	71/51/9	34/20/4	1.196(0.609-2.351)	0.603
Family history of cancer				
No	551/340/53	587/260/39	**1.395(1.147-1.696)**	**0.001**
Yes	45/17/4	38/27/3	0.627(0.284-1.382)	0.247

[a] Sample size of subjects with major homozygote/heterozygote/minor homozygote;
[b] Adjusted for age, sex, BMI, smoking status, drinking status, hypertension, diabetes and family history of cancer in logistic regression Dominant (TC/CC vs. TT) model;
OR, odds ratio; CI, confidence interval.

Table S3. Stratification analyses between *MAGE-A11* rs6641352 polymorphism and clinicopathologic parameters of RCC in dominant model.

Variables	Genotypes		TC/CC vs. TT	
	TT, N (%)	TC/CC, N (%)	OR (95%CI) [a]	P[a]
Controls (N=954)	625 (65.5)	329 (34.5)	1.00 (reference)	
Cases (N=1010)	596 (59.0)	414 (41.0)	**1.315(1.089-1.588)**	**0.004**
Clinical stage				
Localized (I/II)	506 (59.1)	350 (40.9)	**1.309 (1.075-1.594)**	**0.007**
Advanced (III/IV)	90 (58.4)	64 (41.6)	1.409 (0.982-2.061)	0.062
Tumor grade				
Well differentiated (I+II)	434 (59.0)	301 (41.0)	**1.314 (1.071-1.612)**	**0.009**
Moderately differentiated (III)	129 (61.1)	82 (38.9)	1.219 (0.884-1.681)	0.227
Poorly differentiated (IV)	33 (51.6)	31 (48.4)	**1.921 (1.137-3.247)**	**0.015**
Histology				
Clear cell	500 (59.3)	343 (40.7)	**1.271 (1.043-1.549)**	**0.017**
Others	96 (57.5)	71 (42.5)	**1.588 (1.103-2.200)**	**0.012**

[a] Adjusted for age, sex, BMI, smoking status, drinking status, hypertension, diabetes and family history of cancer in logistic regression Dominant (TC/CC vs. TT) model;
OR, odds ratio; CI, confidence interval.

Table S4. Associations between the *MAGE-A11* rs6641352 genotype in dominant model and clinical features of RCC.

Variables	Genotypes		TC/CC vs. TT	
	TT, N (%)	TC/CC, N (%)	OR (95%CI) [a]	P[a]
Clinical stage				0.981[b]
I	387 (58.8)	271 (41.2)	1.000 (reference)	
II	119 (60.1)	79 (39.9)	0.961 (0.677-1.364)	0.824
III	42 (57.5)	31 (42.5)	0.968 (0.568-1.649)	0.904
IV	48 (59.3)	33 (60.7)	0.819 (0.461-1.453)	0.495
Tumor grade				0.493[b]
I	126 (57.0)	95 (43.0)	1.000 (reference)	
II	308 (59.9)	206 (40.1)	0.873 (0.625-1.219)	0.425
III	129 (61.1)	82 (38.9)	0.861 (0.560-1.325)	0.497
IV	33 (51.6)	31 (48.4)	1.332 (0.669-2.650)	0.414
Histology				0.385[b]
Clear cell	500 (59.3)	343 (40.7)	1.000 (reference)	
Papillary	19 (52.8)	17 (47.2)	1.331 (0.672-2.638)	0.413
Chromophobe	28 (50.9)	27 (49.1)	1.595 (0.906-2.806)	0.106
Unclassified	49 (64.5)	27 (35.5)	0.816 (0.483-1.381)	0.449

[a] Adjusted for age, sex, BMI, smoking status, drinking status, hypertension, diabetes, family history of cancer, clinical stage, tumor grade and histology in logistic regression Dominant (TC/CC vs. TT) model;

[b] *P* for χ^2-test of R-by-C table.

OR, odds ratio; CI, confidence interval.

Table S5. Stratification analyses between *MAGE-A11* rs6540341 polymorphism and risk factors in dominant model.

Variables	Cases[a]	Controls[a]	OR (95%CI)[b]	P[b]
Age				
≤57	287/191/50	352/178/30	1.405 (1.095-1.804)	0.008
＞57	274/168/40	234/137/23	1.159 (0.878-1.529)	0.297
BMI				
≤24	290/177/44	313/168/28	1.246 (0.962-1.614)	0.096
＞24	271/182/46	273/147/25	1.376 (1.054-1.797)	0.019
Gender				
Male	358/228/55	382/216/41	1.207 (0.959-1.519)	0.108
Female	204/131/35	204/99/12	**1.483 (1.083-2.029)**	**0.014**
Smoking status				
Never	356/219/63	394/211/34	**1.266 (1.010-1.587)**	**0.041**
Former	96/42/17	40/17/6	1.107 (0.586-2.093)	0.754
Current	110/98/10	152/87/13	**1.516 (1.036-2.218)**	**0.032**
Drinking status				
Never	410/263/68	432/234/38	**1.280 (1.034-1.585)**	**0.024**
Ever	151/96/22	154/81/15	1.315 (0.903-1.914)	0.153
Hypertension				
No	344/221/55	438/230/40	**1.319 (1.055-1.649)**	**0.015**
Yes	217/138/35	148/85/13	1.265 (0.906-1.767)	0.168
Diabetes				
No	491/313/75	548/298/50	**1.255 (1.035-1.522)**	**0.021**
Yes	70/46/15	38/17/3	**2.065 (1.034-4.125)**	**0.04**
Family history of cancer				
No	523/337/84	546/293/47	**1.323 (1.093-1.602)**	**0.004**
Yes	39/22/6	40/22/6	0.928 (0.425-2.206)	0.851

[a]Sample size of subjects with major homozygote/heterozygote/minor homozygote;
[b]Adjusted for age, sex, BMI, smoking status, drinking status, hypertension, diabetes and family history of cancer in logistic regression Dominant (TC/CC vs. TT) model;
OR, odds ratio; CI, confidence interval.

Table S6. Stratification analyses between *MAGE-A11* rs6540341 polymorphism and clinicopathologic parameters of RCC in dominant model.

Variables	Genotypes		CT/TT vs. CC	
	CC,N(%)	TC/TT,N(%)	OR(95%CI)[a]	P[a]
Controls (N=954)	586 (61.4)	368 (38.6)	1.000 (reference)	
Cases (N=1010)	561 (55.5)	449 (44.5)	1.301 (1.081-1.564)	0.005
Clinical stage				
Localized (I/II)	473 (55.3)	383 (44.7)	**1.322 (1.090-1.603)**	**0.005**
Advanced (III/IV)	88 (57.1)	66 (42.9)	1.226 (0.860-1.748)	0.260
Tumor grade				
Well differentiated (I+II)	390 (53.1)	345 (46.9)	**1.430 (1.171-1.747)**	**<0.001**
Moderately differentiated (III)	130 (61.6)	81 (38.4)	1.054 (0.768-1.448)	0.743
Poorly differentiated (IV)	41 (64.1)	23 (35.9)	0.902 (0.528-1.541)	0.705
Histology				
Clear cell	467 (55.4)	376 (44.6)	**1.299 (1.070-1.576)**	**0.008**
Others	94 (56.3)	73 (43.7)	1.307 (0.930-1.836)	0.123

[a] Adjusted for age, sex, BMI, smoking status, drinking status, hypertension, diabetes and family history of cancer in logistic regression Dominant (TC/CC vs. TT) model;
OR, odds ratio; CI, confidence interval.

Table S7. Associations between the *MAGE-A11* rs6540341 genotype in dominant model and clinical features of RCC.

Variables	Genotypes		CT/TT vs. CC	
	CC,N(%)	TC/TT,N(%)	OR(95%CI)[a]	P[a]
Clinical stage				0.941[b]
I	366 (55.6)	292 (44.4)	1.000 (reference)	
II	107 (54.0)	91 (46.0)	1.192 (0.844-1.684)	0.318
III	41 (56.2)	32 (43.8)	1.256 (0.740-2.129)	0.398
IV	47 (58.0)	34 (42.0)	1.243 (0.708-2.184)	0.449
Tumor grade				0.070[b]
I	120 (54.3)	101 (45.7)	1.000 (reference)	
II	270 (52.5)	244 (47.5)	1.022 (0.736-1.418)	0.898
III	130 (61.6)	81 (38.4)	0.659 (0.430-1.010)	0.055
IV	41 (64.1)	23 (35.9)	0.544 (0.271-1.091)	0.086
Histology				0.854[b]
Clear cell	467 (55.4)	376 (44.6)	1.000 (reference)	
Papillary	18 (50.0)	18 (50.0)	1.233 (0.627-2.424)	0.544
Chromophobe	32 (58.2)	23 (41.8)	0.922 (0.523-1.624)	0.778
Unclassified	44 (57.9)	32 (42.1)	1.059 (0.639-1.754)	0.825

[a] Adjusted for age, sex, BMI, smoking status, drinking status, hypertension, diabetes, family history of cancer, clinical stage, tumor grade and histology in logistic regression Dominant (TC/CC vs. TT) model;

[b] *P* for χ^2-test of R-by-C table.

OR, odds ratio; CI, confidence interval.

Table S8. Univariate Cox regression analyses for survival by characteristics and clinical features of RCC patients.

Variables	Patients/death	5-year survival (%)[a]	Log-rank P	HR (95% CI)[b]	P[b]
Total	308/32	80			
Age (year) (mean ± SD)	57.55 ± 11.356		**0.047**		
≤57	158/12	85		1.000 (reference)	
＞57	150/20	73		2.042 (0.994-4.195)	0.052
BMI (kg/m^2) (mean ± SD)	24.22 ± 2.887				
≤24	140/20	76	0.053	1.000 (reference)	
＞24	168/12	83		0.498 (0.243-1.022)	0.057
Gender			0.401		
Male	203/22	78		1.000 (reference)	
Female	105/10	83		0.726 (0.343-1.537)	0.403
Smoking status			**0.026**		
Never	192/15	83		1.000 (reference)	
Ever	116/17	74		**2.162 (1.078-4.337)**	**0.030**
Drinking status			**0.013**		
Never	219/18	86		1.000 (reference)	
Ever	89/14	62		**2.395 (1.17-4.872)**	**0.016**
Hypertension			**0.013**		
No	187/14	85		1.000 (reference)	
Yes	121/18	70		**2.382 (1.180-4.809)**	**0.015**
Diabetes			0.940		
No	262/28	80		1.000 (reference)	
Yes	46/4	75		0.960 (0.336-2.748)	0.940
Family history of cancer			0.443		
No	283/29	80		1.000 (reference)	
Yes	25/3	——		1.589 (0.482-5.224)	0.447
Clinical stage			0.863		
Localized (I/II)	251/25	80		1.000 (reference)	
Advanced (III/IV)	57/7	80		1.077 (0.465-2.492)	0.863
Tumor grade			0.099		
Well differentiated (I+II)	207/18	81		1.000 (reference)	

Moderately differentiated (III)	72/7	82		0.985 (0.411-2.361)	0.973
Poorly differentiated (IV)	29/7	68		**2.439 (1.013-5.874)**	**0.047**
Histology			**0.001**		
Clear cell	256/19	84		1.000 (reference)	
Others	52/13	62		**3.270 (1.612-6.634)**	**0.001**

[a] Proportion of survival derived from Kaplan-Meier analysis.
[b] HR, hazard ratio, calculated by Cox Regression analysis.

Table S9. Stratification analyses of *MAGE-A11* rs6641352 genotypes associated with RCC patients' survival.

Variables	Patients/Death		Log-rank	CC vs. TC/TT	
	TC/TT	CC	P	HR(95%CI)[a]	P[a]
Total	295/26	13/6	**<0.001**	**3.526 (1.072-11.595)**	**0.038**
Age (years)					
≤57	153/10	5/2	**0.033**	7.600 (0.352-164.246)	0.196
＞57	142/16	8/4	**0.005**	3.532 (0.719-17.363)	0.120
BMI (kg/m^2)					
≤24	132/17	8/3	0.079	4.838 (0.909-25.750)	0.065
＞24	163/9	5/3	**<0.001**	39.347 (0.114-13584.833)	0.218
Gender					
Male	192/16	11/6	**<0.001**	**11.565 (2.374-56.346)**	**0.002**
Female	103/10	2/0	0.764	——	0.995
Smoking status					
Never	187/14	5/1	0.278	1.838 (0.163-20.755)	0.623
Ever	108/12	8/5	**0.002**	**116.422 (7.487-1810.333)**	**0.001**
Drinking status					
Never	210/16	9/2	0.189	1.808 (0.278-11.775)	0.536
Ever	85/10	4/4	**<0.001**	**14.442 (1.452-143.653)**	**0.023**
Hypertension					
No	180/11	7/3	**0.002**	**25.071 (2.548-246.701)**	**0.006**
Yes	115/15	6/3	**0.044**	1.980 (0.271-14.465)	0.501
Diabetes					
No	250/22	12/6	**<0.001**	**2.889 (0.849-9.829)**	**0.090**
Yes	45/4	1/0	0.581	1.000 (0.001-666.841)	1.000
Family history of cancer					
No	273/24	10/5	**<0.001**	**6.077 (1.575-23.451)**	**0.009**
Yes	22/2	3/1	0.567	——	——
Clinical stage					
Localized (I/II)	241/20	10/5	**<0.001**	**3.339 (0.866-12.866)**	**0.080**
Advanced (III/IV)	54/6	3/1	0.458	0.057 (0.000-69.339)	0.429
Tumor grade					

Well differentiated (I+II)	198/14	9/4	**0.001**	4.028 (0.764-21.244)	0.101
Moderately differentiated (III)[b]	71/6	1/1	**<0.001**	21440.667(0.001-6.001E+11)	0.254
Poorly differentiated (IV)[b]	26/6	3/1	0.921	0.000(0.000-3.939E+54)	0.737
Histology					
Clear cell	246/15	10/4	**0.001**	5.403 (0.977-29.873)	0.053
Others	49/11	3/2	0.101	5.632 (0.081-391.652)	0.425

[a] Adjusted for age, sex, BMI, smoking status, drinking, hypertension, diabetes, family history of cancer, clinical stage, grade and histology in Cox regression Recessive (CC vs. TC/TT) model.

Table S10. Stepwise Cox regression analysis on RCC-related survival.

Final variables	β	SE	HR	95% CI	P
BMI	-0.226	0.075	0.798	0.689-0.924	0.002
Drinking status	0.482	0.186	1.620	1.125-2.333	0.010
Hypertension	0.846	0.262	2.330	1.394-3.893	0.001
Tumor grade	0.450	0.259	1.568	0.943-2.607	0.083
Clinical stage	-0.519	0.236	0.595	0.375-0.946	0.028
Histology	0.624	0.143	1.866	1.411-2.467	<0.001
rs6641352 (CC vs. TC/TT)	1.846	0.473	6.334	1.917-15.419	<0.001

β, regression coefficient; HR: hazard ratio; CI, confidence interval.

Table S11. Associations between the rs6540341in MAGE-A11 gene and RCC patients' survival.

rs6540341(C/T)	Patients/deaths	5-year survival (%)[a]	Log-rank P	HR (95% CI)	HR (95% CI)[b]	P[b]
Total number of subjects	308/32	80				
Codominant model						
CC	198/19	76		1.000 (reference)	1.000 (reference)	
TC	93/10	83	0.803	0.922 (0.425-2.002)	0.914 (0.367-2.280)	0.848
TT	17/3	——	0.795	1.251 (0.365-4.293)	0.904 (0.193-4.230)	0.898
P trend			0.894			
Additive model			0.898	1.038 (0.597-1.805)	0.937 (0.475-1.848)	0.851
Dominant model						
CC	198/19	76		1.000 (reference)	1.000 (reference)	
TC/TT	110/13	83	0.957	0.980 (0.478-2.009)	0.913 (0.379-2.201)	0.839
Recessive model						
CC/TC	291/29	79		1.000 (reference)	1.000 (reference)	
TT	17/3	——	0.677	1.290 (0.389-4.279)	0.951 (0.222-4.079)	0.946

BMI, body mass index; HR, hazard ratio.

Values in bold indicate statistically different.

[a] Proportion of survival derived from Kaplan-Meier analysis.

[b] Adjusted for age, sex, BMI, smoking status, drinking status, hypertension, diabetes and family history of cancer, clinical stage, tumor stage and histology in Cox regression model.
